# Development and evaluation of a SYBR Green real-time RT-PCR assay for detection of avian hepatitis E virus

**DOI:** 10.1186/s12917-015-0507-5

**Published:** 2015-08-11

**Authors:** Qin Zhao, Sha Xie, Yani Sun, Yiyang Chen, Jiming Gao, Huiya Li, Xinjie Wang, Shahid Faraz Syed, Baoyuan Liu, Lizhen Wang, Gaiping Zhang, En-Min Zhou

**Affiliations:** Department of Preventive Veterinary Medicine, College of Veterinary Medicine, Northwest A&F University, Yangling, Shaanxi 712100 China; College of Animal Science and Veterinary Medicine, Henan Agricultural University, Zhengzhou, Henan 450002 China

**Keywords:** Avian hepatitis E virus, Diagnosis, SYBR Green real-time RT-PCR

## Abstract

**Background:**

Avian hepatitis E virus (HEV) is the main causative agent of big liver and spleen disease, as well as hepatitis-splenomegaly syndrome in chickens. To date, conventional reverse transcriptase polymerase chain reaction (RT-PCR) and nested RT-PCR methods have been used for the diagnosis of avian HEV infection in chickens. However, these assays are time consuming, inconvenient, and cannot detect the virus quantitatively. In this study, a rapid and sensitive SYBR Green real-time RT-PCR assay was developed to detect avian HEV RNA quantitatively in serum, liver, spleen, and fecal samples from chickens.

**Results:**

Based on the sequence of the most conserved HEV gene, ORF3, the primers for the assay were designed, and the standard plasmid was constructed. The detection limit of the assay was shown to be 10 copies/μl of standard plasmid/reaction, with a corresponding cycle-threshold value of 29.3. The standard curve exhibited a dynamic linear range across at least 7 log units of DNA copy number. The specificity and reproducibility of this assay was high, showing that the assay detected avian HEV RNA specifically and with little variability. Compared to conventional RT-PCR, the current assay is more sensitive for detecting avian HEV in serum, liver, spleen, and fecal samples from chickens.

**Conclusions:**

A rapid, specific, and reproducible SYBR Green real-time RT-PCR assay was developed for the diagnosis of avian HEV infection in chickens. This assay can accurately detect avian HEV RNA in serum, liver, spleen, and fecal samples with more sensitivity than conventional RT-PCR.

## Background

Avian hepatitis E virus (HEV) is the main causative agent of big liver and spleen disease, as well as hepatitis-splenomegaly syndrome in chickens [1–3]. Both of these diseases result in a decrease in egg production, an increase in mortality, and an enlargement of liver and spleen in broiler breeders and egg laying hens [4–7]. In addition, the antibodies and viral RNA of avian HEV were universally detected in healthy chicken flocks, indicating that the virus can cause subclinical infections in chickens [8–11].

Avian HEV is a non-enveloped, positive-sense, single-stranded RNA virus, belonging to the family *Hepeviridae* along with human and swine HEVs [12]. The complete genome of avian HEV is approximately 6.6 kb and shares roughly 50 % nucleotide sequence identity with human and swine HEVs [12–15]. Similar to human and swine HEVs, the genome of avian HEV contains two noncoding regions and three open reading frames (ORFs). ORF1 encodes a non-structural protein, ORF2 encodes a capsid protein, and ORF3 (overlapping partially with ORF2), encodes a small phosphoprotein [12, 13]. Based on phylogenetic analysis, avian HEV forms a separate genus from mammalian HEV and is divided into at least three different genotypes [15, 16]. The genomes of the different genotypes share approximately 80 % nucleotide sequence identity, while there is over 90 % nucleotide sequence identity within a single genotype [14, 15].

Currently there is not an efficient cell culture system for propagating avian HEV. Avian HEV infection is generally diagnosed using ELISA to test for the presence of antibodies, and RT-PCR to detect viral RNA [8, 16–19]. To date, some conventional RT-PCR and nested RT-PCR methods have been developed for the diagnosis of avian HEV infection [8, 17]. Among these methods, the RT-PCR assays developed by Huang et al. were widely used for detection avian HEV infection in the flocks from the different countries [17]. However, these assays are time consuming, cumbersome, and prone to falsely positive results due to cross-contamination. Compared to conventional RT-PCR, real-time RT-PCR (using SYBR Green and TaqMan) has many advantages, including simplicity, shorter detection times, and lower contamination rates, as well as higher sensitivity and specificity rates. This assay is also high throughput and easily standardized. One TaqMan real-time RT-PCR assay has been previously described for the detection and quantification of avian HEV in clinical samples [20]. In comparison with a TaqMan assay, SYBR Green real-time RT-PCR is preferable due to the relatively low cost and simplified primer design. However, there is currently no report validating the use of the SYBR Green real-time RT-PCR for the detection and quantification of avian HEV RNA.

The aim of the present study was to develop a rapid, sensitive, and specific SYBR Green real time RT-PCR assay for the identification and quantification of avian HEV RNA in chicken samples and to compare its sensitivity and specificity with the currently prevailing RT-PCR methods which were developed by Huang et al. and widely used for detection avian HEV RNA in clinical samples from the chickens in different countries.

## Results

### Preparation of the standard plasmid

The 264 base pair (bp) ORF3 gene from a Chinese avian HEV strain (CaHEV) was amplified by RT-PCR and cloned into the commercial clone vector pMD 18-T (TaKaRa Biotech Corporation, Dalian, China). Then, the recombinant plasmid was prepared for the standard control plasmid. The inserted fragment was sequenced and confirmed for 100 % nucleotide identity with the expected ORF3 sequence of CaHEV. The concentration of the recombinant plasmid was 42.5 ng/μl, and the copy number was 1.30 × 10^10^ copies/μl.

### Detection limit and standard curve of the assay

**Fig. 1:**
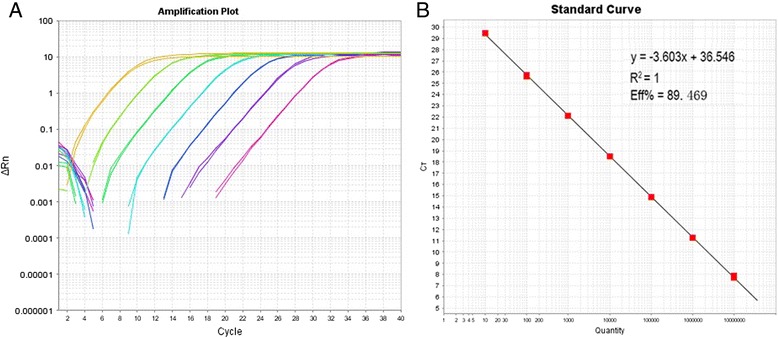
Detection of 10-fold serial dilutions (10^7^ to 10^1^ copies/μl) of standard plasmid using the SYBR Green real-time RT-PCR. **a**: Avian HEV SYBR Green real-time RT-PCR amplification plot. **b**: Standard curve where each dot represents the cycle threshold value for the amplified standard plasmid at a given copy number

The detection limit of the SYBR Green real-time PCR assay was determined by testing 10-fold serial dilutions of the standard plasmids (10^7^ to 10^1^ copies/μl) in duplicate. The minimum limit of detection was shown to be 10 copies/μl of standard plasmids/reaction, with a corresponding cycle-threshold (Ct) value of 29.3 (Fig. 1a). By plotting the Ct values against the copy numbers of the diluted standard plasmid DNA, the standard curve exhibited a dynamic linear range across at least 7 log units of DNA copy number (10^7^ to 10^1^ copies/μl). Linear regression analysis revealed that the efficiency of the assay was 0.895 and the correlation coefficient (R^2^) was 1, with a slope value of 3.60 (Fig. 1b).

### Specificity and reproducibility of the assay

**Fig. 2:**
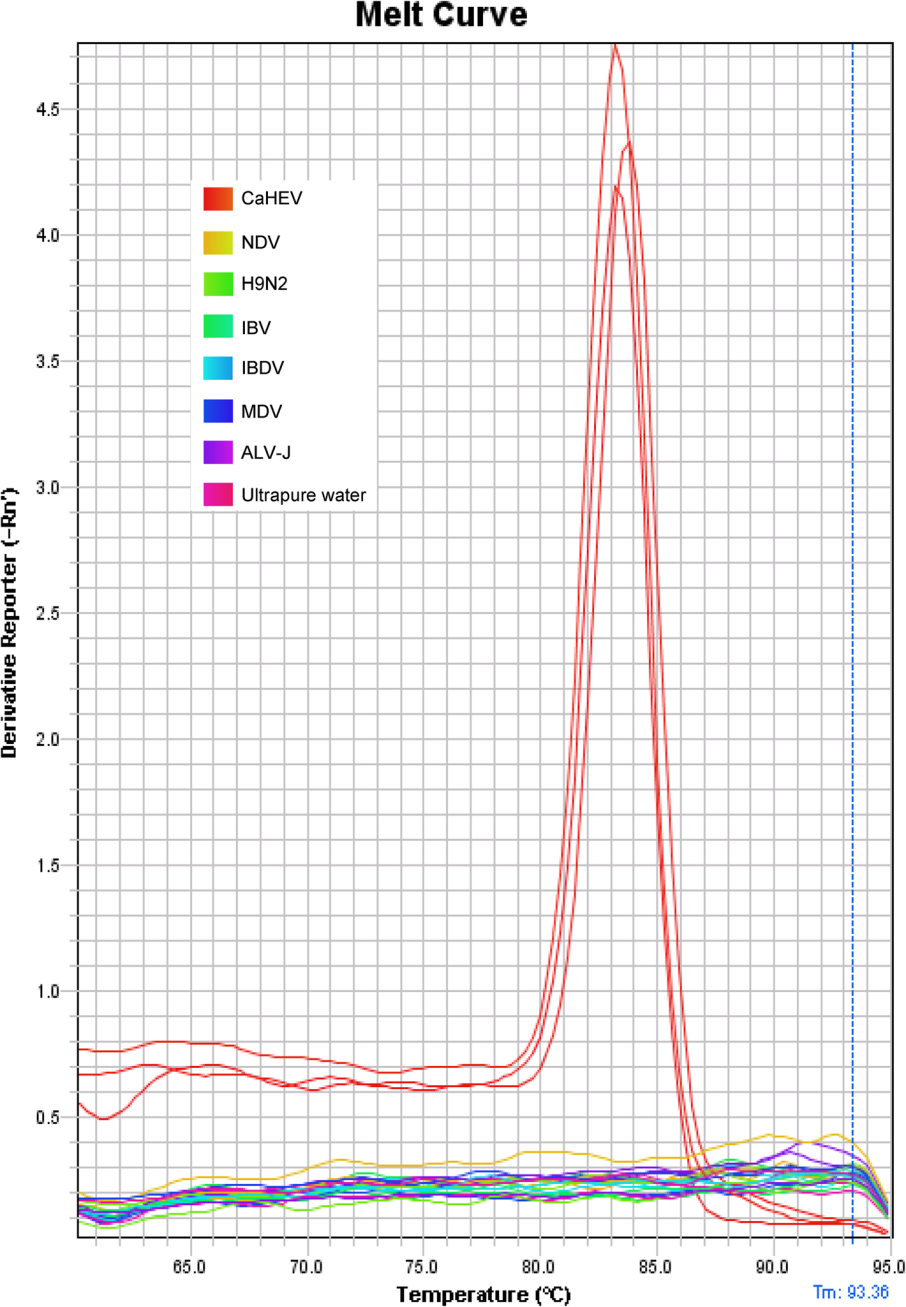
Specificity of the SYBR Green real-time RT-PCR assay. DNA or RNA from CaHEV and six other avian viruses were tested in triplicate using the SYBR Green real-time RT-PCR assay

The specificity of the SYBR Green real-time RT-PCR assay was determined by testing the following 6 avian viruses in triplicate: Newcastle disease virus (NDV), avian influenza virus (H9N2), infectious bronchitis virus (IBV), infectious bursal disease virus (IBDV), Marek’s disease virus (MDV), and J group of avian leucosis virus (ALV-J). In addition, the CaHEV stock and ultrapure water were used as positive and negative controls, respectively. In the assay only CaHEV was detected as a single melt peak and the other six viruses and negative control were not found (Fig. 2). The mean Ct value of CaHEV was 26.8 and no Ct values were measured for the other viruses or the ultrapure water.

The reproducibility of the assay was determined by testing five 10-fold serial dilutions of standard plasmids (10^−1^ to 10^−5^) in triplicate using inter- and intra-assay comparisons. For the standard plasmids, using dilutions from 10^−1^ to 10^−5^, the values of the intra-assay standard deviation (SD) and coefficient of variation (CV) ranged from 0.050 to 0.33 and 0.23 % to 1.6 %, respectively, and the values of the inter-assay SD and CV ranged from 0.070 to 1.3 and 0.32 % to 5.4 %, respectively (Table 1). These data indicated that the assay was repeatable and reproducible with low variation.

**Table 1:** Reproducibility of intra- and inter-assay with different dilution standard plasmids by SYBR Green real-time RT-PCR

Variation	Different dilutions	Ct values for the different dilution of standard plasmids				
		1	2	3	Mean Ct ± SD	CV (%)
Inter-assay	10^−5^	27.91	28.23	28.07	28.07 ± 0.16	0.58
	10^−4^	23.24	25.67	25.38	24.76 ± 1.33	5.37
	10^−3^	21.89	21.97	22.03	21.96 ± 0.07	0.32
	10^−2^	17.25	18.12	18.23	17.87 ± 0.54	3.02
	10^−1^	14.10	14.02	14.97	14.36 ± 0.53	3.69
Intra-assay	10^−5^	27.38	27.54	28.01	27.64 ± 0.33	1.19
	10^−4^	25.10	25.23	24.98	25.10 ± 0.13	0.52
	10^−3^	22.07	22.16	22.13	22.12 ± 0.05	0.23
	10^−2^	17.98	18.12	18.05	18.05 ± 0.07	0.39
	10^−1^	14.54	14.78	15.01	14.78 ± 0.24	1.62

### Detection of viral RNA in sequential serum samples from CaHEV challenged chickens

**Fig. 3:**
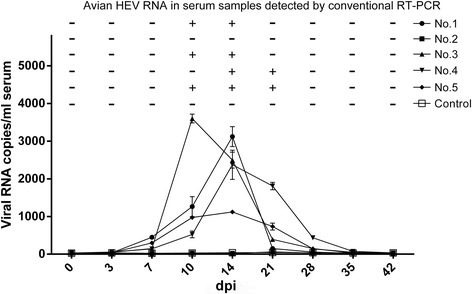
SYBR Green real-time RT-PCR and conventional RT-PCR detection of HEV viral RNA in serum samples from chickens challenged with CaHEV. In the linear graph (*bottom*), viral RNA in serum samples was quantitatively detected using the SYBR Green real-time RT-PCR. Each data point represents the mean value (±SD) generated from three replicates. The line graph plotted above depicts qualitative detection of avian HEV RNA by conventional RT-PCR. A “-” represents a negative sample and a “+” represents a positive sample

Chickens were challenged with CaHEV, and sequential serum samples were collected at 0, 3, 7, 10, 14, 21, 28, 35, and 42 days post-inoculation (dpi) and analyzed for the presence of viral RNA using SYBR Green real-time RT-PCR and conventional RT-PCR. Four of the five challenged chickens were observed with viremia. Using SYBR Green real-time RT-PCR, viral RNA was detected in the infected chickens starting at 7 dpi and ending at 28 dpi. Conventional RT-PCR detected viral RNA in samples from infected chickens starting at 10 dpi and ending at 21 dpi (Fig. 3). The highest amount of viral RNA detected was in the sera of chicken No. 3 at 10 dpi, with a mean titre of 3601 copies/ml. Chickens No. 1, 4, and 5 showcased their viral RNA peak at 14 dpi, with titres ranging from 1120–3121 copies/ml (Fig. 3). No viral RNA was detected in the five chickens from the control group or in chicken No. 2 from the challenged group (Fig. 3).

### Comparison of SYBR Green real-time RT-PCR and conventional RT-PCR for sensitivity in detecting positive clinical samples

SYBR Green real-time RT-PCR and conventional RT-PCR were performed simultaneously to test 90 clinical samples (32 livers, 16 spleens, and 42 fecal samples), which were collected at different time intervals from birds showing a decrease in egg production in the Shaanxi province of China. Of the 90 samples collected, 22 samples (7 livers, 4 spleens, and 11 fecal samples) were found to be HEV positive using conventional RT-PCR (Table 2). However, 11 additional samples (4 livers and 7 fecal samples) were identified to be positive by SYBR Green real-time RT-PCR (Table 2). These additional samples were confirmed to be positive using sequence analysis (Data not shown). These data indicated that SYBR Green real-time RT-PCR can detect avian HEV RNA in clinical samples with more sensitivity than conventional RT-PCR.

**Table 2:** Detection of the clinical samples from the chickens with a decrease in egg production by SYBR Green real-time RT-PCR and conventional RT-PCR

Types of samples	Total number	Positive number for conventional RT-PCR	Positive number for SYBR Green real-time RT-PCR
Liver	32	7	11
Spleen	16	4	4
Faeces	42	11	18
Total	90	22	33

## Discussion

Over the last two decades remarkable progress has been made in the application of RT-PCR for the diagnosis of avian HEV infection in chickens [8, 17, 18]. However there are currently no efficient cell culture lines available for the propagation of HEV. Several conventional RT-PCR systems have been used for the detection of partial ORF2 and ORF1 genes from avian HEV in various biological samples such as fecal matter, serum, bile, and liver [9, 16–18]. However, these conventional RT-PCR assays are time consuming and labor-intensive with a high risk of contamination. Compared with conventional RT-PCR, the real-time RT-PCR assay is a more rapid and sensitive test and is less likely to produce false positive by contamination during sample preparation. Recently, a more rapid and sensitive TaqMan real-time RT-PCR assay has been reported and are currently under extensive evaluation with clinical chicken samples including livers, fecal and serum samples [20]. However, since the TaqMan real-time RT-PCR assay uses the 5’-3’ nuclease oligoprobe, it is not economical to perform routine testing. As an alternative, the SYBR Green real-time RT-PCR assay can be applied directly to any gene without the need to design and synthesize fluorescently labeled target-specific probes. In addition, the SYBR Green fluorogenic molecules is less expensive, simple in primer design and its universal RT-PCR protocols are suitable for multiple target sequences than the 5’-nuclease TaqMan assay. In this study, a more rapid, simple to operate, and sensitive SYBR Green real-time RT-PCR assay was developed and evaluated for its accuracy and sensitivity in detecting the avian HEV RNA in chicken fecal, liver, spleen, and serum samples.

The RT-PCR assays which have been established in the past use almost exclusively primers designed to target the ORF1 and ORF2 genes of avian HEV. However, sequence alignment showed that the ORF3 gene is actually the most conserved region of the avian HEV genome. Consequently, the SYBR Green real-time PCR assay developed in this study used primers targeting ORF3 gene. In addition, based on sequence alignment, the 3’ terminal nucleotide sequences used to create these primers are the same in different genotypes of avian HEV strains. Based on this information this SYBR Green real-time RT-PCR assay may be used for the universal detection of avian HEV.

To compare the SYBR Green real-time RT-PCR with conventional RT-PCR assays, sequential serum samples were obtained from chickens challenged with CaHEV, and clinical samples were obtained from chickens with a decrease in egg production. These samples were tested simultaneously with the two assays. For the sequential serum samples, viral RNA was detected as a sign of infection. The SYBR Green real-time RT-PCR assay proved to be more sensitive than conventional RT-PCR, as viral RNA was detected over a larger range of time during the progression of infection compared to conventional RT-PCR (Fig. 3). For the 90 clinical samples, only 22 samples were found to be positive using conventional RT-PCR, while 33 were identified as positive using SYBR Green real-time RT-PCR (Table 2). These present data indicated that this newly validated assay is more sensitive than the conventional RT-PCR for early detection and surveillance of avian HEV infection in chickens. This development will be economically important for the poultry industry in the prevention of losses due to HEV infection.

## Conclusions

A SYBR Green real-time RT-PCR assay for the detection and quantification of avian HEV RNA was developed. This assay was proven to be efficient, specific, reproducible, and more sensitive than conventional RT-PCR in the detection of avian HEV RNA from serum, liver, spleen, and fecal samples. Effective disease management relies on diagnostic kits to confirm viral infection early and accurately. In this regard, the commercial availability of this assay may be useful for the diagnosis and management of avian HEV infection in poultry farms.

## Methods

### Virus strain

The CaHEV stock used in this study was prepared from bile suspensions collected from a chicken with hepatitis-splenomegaly syndrome in the Shandong province of China (GenBank accession number: GU954430) [15].

### Chickens and samples collection

Five 8-week-old specific-pathogen-free (SPF) chickens (Beijing Merial Vital Laboratory Animal Technology Co., Beijing, China) were housed in an isolation facility and were inoculated intravenously with 0.8 ml of CaHEV stock (10^4^ genomic amount/ml). A group of uninfected chickens (*n* = 5) were kept separately as negative controls. Sequential serum samples (200 μl) were collected from these chickens at 0, 3, 7, 10, 14, 21, 28, 35, and 42 dpi. In addition, a set of clinical samples from liver (*n* = 32), spleen (*n* = 16), and feces (*n* = 42), were collected from birds showing a decrease in egg production in the Shaanxi province of China. Each sample was homogenized in 10 % sterile 0.1 M phosphate-buffered saline (PBS, pH = 7.4) and clarified by centrifugation at 3000 rpm for 15 min at 4 °C. Total RNA was extracted from the supernatant. Animal experiments were conducted under the guidelines of the Institutional Animal Care and Use Committee of Northwest A&F University and were approved by the Institutional Animal Care and Use Committee of Northwest A&F University with the approval license number of NWAFU (Shaan) 20120910/12.

### Primer design

The complete genome of CaHEV was aligned with four other avian HEV genomes from GenBank (GenBank accession numbers AM943647, AM943646, AY535004, and EF206691) using the Clustal W method of Lasergene 7.1 MegAlign program (DNASTAR Inc., Madison, WI, USA). As documented in a previous study, the ORF3 gene of avian HEV was the most conserved (Data not shown). The region selected for primer design was based on the nucleotide sequences of the CaHEV ORF3 gene. The F1 primer 5’-CGTGACAACTCAGCCCAGTG-3’ (covering nucleotides 4801–4821), and R1 primer 5’-GCGGTGACAACGTCGGTA-3’ (complementary to nucleotides 4866–4883), were used for real-time amplification of avian HEV. The F2 primer 5’-ATGTATCTTAGTTGCCAGTTCTGG-3’ (covering nucleotides 4654–4682), and R2 primer 5’-CTACATCTGGTACCGTG-3’ (complementary to nucleotides 4900–4917), were designed for the construction of the standard plasmid.

### Viral RNA extraction and reverse transcription

Total RNA was extracted from the CaHEV stock, serum, liver, spleen, and fecal samples using RNAiso Plus reagents according to the manufacturer’s instructions (TaKaRa Biotech Corporation, Dalian, China). RNA samples were then dissolved in 16 μl of DNase-, RNase-, and Proteinase-free water. The first strand of cDNA was synthesized using the Reverse Transcriptase M-MLV (RNase H^−^) Kit (TaKaRa Biotech Corporation, Dalian, China). The reaction volume was as follows: 2 μl 5×M-MLV buffer, 0.5 μl dNTP mix (10 mmol/L), 1 μl Random 6 mers (50 μM), 0.25 μl RNase Inhibitor (40 U/μl), 0.25 μl M-MLV (200 U/μl), and 6 μl of RNA. The reaction was performed at 42 °C for 30 min, and finished at 80 °C for 15 s.

### Standard plasmid DNA

A fragment containing the complete sequence of the CaHEV ORF3 gene was amplified using the F2 and R2 primers to prepare the standard control plasmid. The RT-PCR products were then cloned into the pMD 18-T vector according to the manufacturer’s instructions (TaKaRa Biotech Corporation, Dalian, China). The standard plasmid DNA was extracted from transformed *Escherichia coli* DH5α competent cells using the EasyPure Plasmid MiniPrep Kit (Transgen Biotech Corporation, Beijing, China) and confirmed with DNA sequencing (Genscript Biotech Corporation, Nanjing, China). DNA concentration was determined by spectrophotometry (Epoch) at 260 nm, and the average concentration was determined using five independent measurements. The copy number of the recombinant plasmid was calculated using the following formula: (DNA concentration in ng/μl×10^−9^×6.0233×10^23^ copies/mol)/[DNAsize (bp)×660] [21].

### SYBR Green real-time RT-PCR assay

The real-time RT-PCR assay was carried out using the FastStart Universal SYBR Green Master (ROX) Kit (Roche Diagnostics GmbH, Mannheim, Germany). Optimization of primer concentration was achieved using dilutions of RNA (0.1 μM to 0.7 μM) from CaHEV stock. A concentration of 0.5 μM of each primer was found to yield the highest sensitivity. Real-time RT-PCR was performed with a final volume of 10 μl containing 5 μl of 2 × SYBR Green Master Mix, 0.25 μl each of 0.5 μM F1 forward and R1 reverse primers, 2.5 μl cDNA or standard plasmid DNA, and 2 μl of ultrapure water. The optimized thermal cycling conditions were as follows: 95 °C for 5 min, 95 °C for 15 s, and 60 °C for 30 s, for 40 cycles; 95 °C for 30 s, 60 °C for 20 s, and 95 °C for 30 s, for 1 cycle. For each run, an ultrapure water negative control was included. All real-time RT-PCR reactions were conducted using the Step One Plus Real-Time PCR System (Applied Biosystems®, California, USA). The fluorescence was measured at the end of each cycle.

### Detection limit and standard curves

To determine the detection limit of the assay, 10-fold serial dilutions of the standard plasmid, (10^7^ to 10^1^ copies/μl in ultrapure water), were run in duplicate through the SYBR Green real-time RT-PCR assay. The obtained Ct values were plotted against the DNA copy number to construct a standard curve.

### Specificity and reproducibility

To determine the specificity of the assay, RNA or DNA extracted from six other avian viruses (NDV, H9N2, IBV, IBDV, MDV and ALV-J) were tested in triplicate. Avian HEV positive samples and an ultrapure water negative control were included in each run.

To determine the reproducibility of the real-time PCR assay, intra-assay and inter-assay tests were performed using five different 10-fold dilutions (10^−1^ to 10^−5^) of standard plasmid. The intra-assay test was performed in triplicate within the same run, and the inter-assay test was conducted independently as three different runs on different days. The mean, SD, and CV for both the intra-assay and inter-assay tests were calculated separately for each standard DNA dilution based on their Ct values using Microsoft Excel software.

### Conventional RT-PCR

The conventional RT-PCR assays for amplification of avian HEV RNA from serum and clinical samples were performed using a 2720 thermal cycler (Applied Biosystems®, California, USA) as described previously with some optimized modifications [17]. Briefly, the PCR mixture contained 12.5 μl of 2 × Premix Taq (loading mix) (TaKaRa Biotech Corporation, Dalian, China), 1 μl each of forward and reverse primers, 5 μl of the cDNA, and 5.5 μl of ultrapure water. The PCR parameters included a denaturation at 95 °C for 10 min followed by 39 cycles of denaturation for 50 s at 94 °C, annealing for 50 s at 50 °C, and extension for 50 s at 72 °C, with a final incubation at 72 °C for 10 min. The PCR yielded a 372 bp product, which was analyzed by electrophoresis on a 1.0 % agarose gel stained with ethidium bromide.

## Abbreviations

HEV: Hepatitis E virus
ORF: Open reading frame
ELISA: Enzyme-linked immunosorbent assay
RT-PCR: Reverse transcriptase polymerase chain reaction
Ct: Cycle-threshold
SD: Standard deviation
CV: Coefficient of variation
SPF: Specific-pathogen-free
